# Functional Similarities between the Protein *O*-Mannosyltransferases Pmt4 from Bakers' Yeast and Human POMT1*[Fn FN2]

**DOI:** 10.1074/jbc.M116.739128

**Published:** 2016-06-29

**Authors:** Daniela Bausewein, Jakob Engel, Thomas Jank, Maria Schoedl, Sabine Strahl

**Affiliations:** From the Centre for Organismal Studies, Cell Chemistry, Heidelberg University, 69120 Heidelberg, Germany

**Keywords:** enzyme mutation, glycosylation, glycosyltransferase, human, yeast, dystroglycanopathy, O-mannosylation, PMT4, POMT1

## Abstract

Protein *O*-mannosylation is an essential post-translational modification. It is initiated in the endoplasmic reticulum by a family of protein *O*-mannosyltransferases that are conserved from yeast (PMTs) to human (POMTs). The degree of functional conservation between yeast and human protein *O*-mannosyltransferases is uncharacterized. In bakers' yeast, the main *in vivo* activities are due to heteromeric Pmt1-Pmt2 and homomeric Pmt4 complexes. Here we describe an enzymatic assay that allowed us to monitor Pmt4 activity *in vitro*. We demonstrate that detergent requirements and acceptor substrates of yeast Pmt4 are different from Pmt1-Pmt2, but resemble that of human POMTs. Furthermore, we mimicked two POMT1 amino acid exchanges (G76R and V428D) that result in severe congenital muscular dystrophies in humans, in yeast Pmt4 (I112R and I435D). *In vivo* and *in vitro* analyses showed that general features such as protein stability of the Pmt4 variants were not significantly affected, however, the mutants proved largely enzymatically inactive. Our results demonstrate functional and biochemical similarities between POMT1 and its orthologue from bakers' yeast Pmt4.

## Introduction

*O*-Mannosylation of secretory and membrane proteins is a conserved, essential modification in eukaryotes. In yeast, this post-translational modification is important for the biosynthesis and maintenance of the cell wall, and even affects quality control of proteins in the endoplasmic reticulum (ER)^6^ (reviewed in Refs. 1 and 2). In humans, a heterogeneous group of congenital muscular dystrophies, collectively referred to as secondary α-dystroglycanopathies, is connected to the reduced *O*-mannosylation of the cell surface-associated basement membrane receptor α-dystroglycan (αDG) (reviewed in Ref. 3). Furthermore, *O*-mannosyl glycans are also commonly present among members of the cadherin and plexin families and influence cadherin-mediated cell-cell adhesion (4–6).

Biosynthesis of *O*-mannosyl glycans is initiated by a family of protein *O*-mannosyltransferases (PMTs), which is highly conserved among eukaryotes except plants and nematodes (reviewed in Refs. 1 and 7). PMTs catalyze the transfer of mannose from dolichol-phosphate mannose (Dol-P-Man) to the hydroxyl group of serine or threonine residues of proteins in the lumen of the ER. These enzymes are essential for the viability of yeasts, filamentous fungi, and animals (5, 8–12). Numerous mutations in the human PMTs (POMT1, POMT2) have been identified that cause various forms of α-dystroglycanopathies, with Walker-Warburg syndrome (WWS) being the most severe form of these disorders (13–16).

In bakers' yeast, the redundant PMT family is grouped into three subfamilies: PMT1 (Pmt1 and Pmt5), PMT2 (Pmt2, Pmt3, and Pmt6), and PMT4 (Pmt4). The main mannosyltransferase activities are due to Pmt1, Pmt2, and Pmt4 (8, 17). Today, the best studied family member is Pmt1. A topology model comprising seven transmembrane spans and two prominent luminal loops has been established for *Saccharomyces cerevisiae* Pmt1 and is likely conserved in other eukaryotic PMTs (18). An acceptor substrate binding domain has been mapped to Pmt1-loop1 using photoreactive peptides, but amino acids crucial for enzyme function are spread over the entire protein, which hampered attempts to pinpoint the catalytic center via targeted mutagenesis (19, 20). The large hydrophilic Pmt1-loop5 domain is crucial for enzyme function although it is most probably not involved in the basic catalytic mechanism (20). Database mining has revealed that this loop contains three conserved, so called MIR motifs (21), but the function of those, as well as the entire loop5 is undefined.

The yeast PMT1 and PMT2 family members and Pmt4 differ in several aspects. PMT1 and PMT2 mannosyltransferases form distinct heteromeric complexes, whereas, Pmt4 acts as a homomeric complex (22). Mutations of a conserved DE-motif in the loop1 domain that influence protein substrate binding of Pmt1, differentially affect mannosyltransferase activities of Pmt1-Pmt2 and Pmt4 (19). Pmt1-Pmt2 and Pmt4 act on distinct protein substrates *in vivo*, and Pmt4 preferentially modifies membrane-anchored proteins (23, 24). Furthermore, *in vitro* assays suitable to measure the enzymatic activity of Pmt1-Pmt2 did not monitor Pmt4 mannosyltransferase activity, further pointing to distinct acceptor requirements (8). How PMTs recognize their acceptor substrates is still enigmatic although computational and experimental approaches have been conducted to define consensus mannosylation motifs (reviewed in Ref. 1; 25).

In contrast to yeast, in mammals only two PMTs, namely POMT1 and POMT2 are present (reviewed in Ref. 26). However, although Pmt2 is the closest homolog of mammalian POMT2, POMT1 is a homolog of yeast Pmt4, and the PMT1 subfamily is absent in higher eukaryotes. POMT1 and POMT2 have been demonstrated to act as a heteromeric complex, however, when compared with yeast different amino acid residues might govern complex formation (27, 28). Furthermore, it was suggested that the two mammalian proteins contribute differentially to mannosyltransferase activity (28). POMT1-POMT2 act on αDG *in vivo* and *in vitro* (29, 30). But, just like for yeast PMTs, mannosylation motifs are poorly defined.

The molecular analysis of eukaryotic polytopic transmembrane protein *O*-mannosyltransferases is still a challenge to which heteromeric complex formation adds a further level of complexity (19). Thus, homomeric Pmt4 appears to be a promising model to further characterize this essential class of enzymes. In this study we describe *in vitro* properties of Pmt4 from bakers' yeast and show its functional relationship with human POMT1.

## Results

### 

#### 

##### S. cerevisiae Pmt4 but not Pmt1-Pmt2 Complexes Mannosylate the Human POMT Substrate α-Dystroglycan in Vitro

**FIGURE 1.**
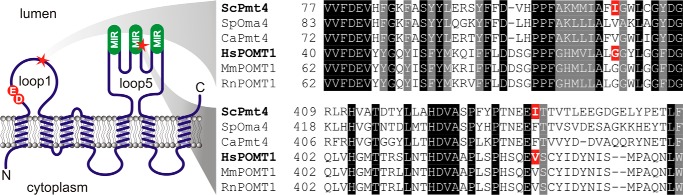
**Conservation of PMT4 family members from fungi and mammals.** Alignment of the regions surrounding the characterized WWS-associated mutants (highlighted in *red*) of PMT4 family proteins from *S. cerevisiae* (*Sc*Pmt4), *S. pombe* (*Sp*Oma4), *Candida albicans* (*Ca*Pmt4), human (*Hs*POMT1), mouse (*Mm*POMT1), and rat (*Rn*POMT1) are shown on the *right*. The location of the amino acid exchanges G76R/I112R and V428D/I435D (*red stars*) is illustrated within a topology model of PMT/POMTs (*left*). The conserved DE motif and the three MIR domains are marked.

**FIGURE 2.**
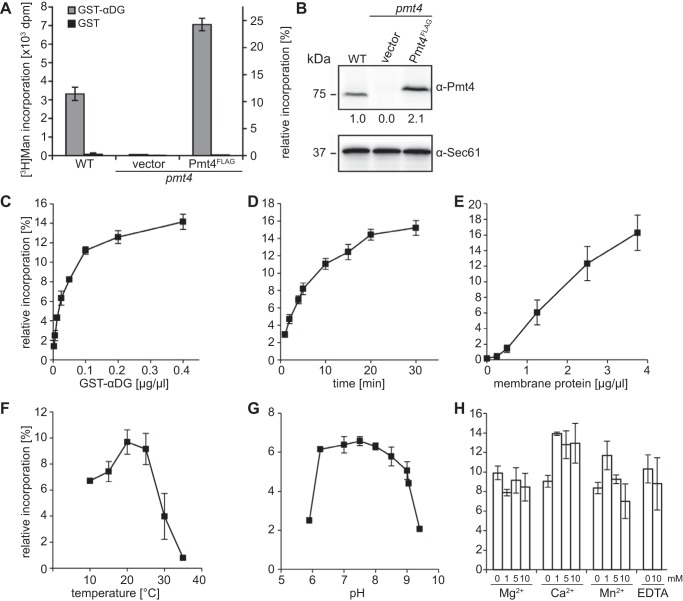
**Pmt4 *in vitro* mannosyltransferase activity.** *A,* crude membranes from wild-type strain SEY6210 (WT) and *pmt4* null mutant transformed with pRS423 (vector) or pJK4-B1 (Pmt4^FLAG^) were tested for *in vitro* mannosyltransferase activity. The standard assay containing 29,067 dpm of Dol-P-[^3^H]Man per reaction was performed as described under ”Experimental Procedures.“ Mean ± S.D. values of at least three independent experiments are shown. *B,* quantification of the Pmt4 content of the membranes used in *A*. Western blottings were probed with anti-Pmt4 and anti-Sec61 antibodies. Signal intensities were quantified as outlined under ”Experimental Procedures.“ The indicated *numbers* represent the relative Pmt4 content (ratio Pmt4:Sec61) with reference to WT, which was set as 1. *C–H,* characterization of the Pmt4 *in vitro* mannosyltransferase activity. Standard assays containing between 20,000 and 30,000 dpm of Dol-P-[^3^H]Man per reaction were performed. Mean ± S.D. values of independent experiments are shown (*n* = 2 to 4). Dependence of the *in vitro* mannosyltransfer reaction on the concentration of the mannose acceptor GST-αDG (*C*), the reaction time (*D*), the membrane protein input (*E*), the reaction temperature (*F*), the pH (*G*), and the presence of divalent cations and EDTA at the depicted concentrations (*H*) was determined using SEY6210 membranes as an enzyme source.

The PMT4 family mannosyltransferases from bakers' yeast (Pmt4) and human (POMT1) show a high degree of conservation (Fig. 1) (26). To establish an *in vitro* assay to monitor Pmt4-mediated mannosyl transfer, we thus tested conditions previously used for *in vitro* activity measurements of the mammalian POMTs (31). Indeed, the use of GST-tagged α-dystroglycan mucin domain (GST-αDG) as acceptor substrate and β-octylthioglucoside (β-OTG) as detergent enabled detection of yeast Pmt4 activity *in vitro* (Fig. 2*A*) (19). In a reaction mixture containing [^3^H]mannose-labeled Dol-P-Man as donor substrate and crude membranes isolated from wild-type yeast strain SEY6210 as enzyme source, typically 10 to 15% of the tritiated mannose were transferred to GST-αDG, but not to GST alone (Fig. 2*A*). Remarkably, this assay exclusively monitored Pmt4 activity because in total membranes from a *pmt4*Δ null mutant strain mannosyltransferase activity was around background level, although all PMT1 and PMT2 family members were present (Fig. 2*A*). Expression of a FLAG-tagged version of Pmt4 (Pmt4^FLAG^) from a multicopy plasmid restored *in vitro* mannosyltransferase activity in the latter strain. The roughly 2-fold increase in mannosyl transfer (Fig. 2*A*) correlated well with 2.1-fold higher enzyme content when compared with wild-type membrane preparations (Fig. 2*B*).

Based on these observations, a standardized Pmt4 *in vitro* mannosyltransferase activity assay was elaborated (see “Experimental Procedures”) using membrane preparations from wild-type yeast, and various parameters were characterized and optimized. Variation of the GST-αDG input yielded a plateau at around 0.1 μg/μl validating that the acceptor substrate was not limiting at a concentration of 0.2 μg/μl, which was routinely used in standard reactions (Fig. 2*C*). Time course experiments revealed a plateau of the reaction after ∼15 min, although a significant amount of the [^3^H]mannose was not detected on GST-αDG at that time (Fig. 2*D*). For up to 2 μg/μl of total membrane protein the incorporation of [^3^H]mannose increased proportional to the enzyme input (Fig. 2*E*). Temperatures above 25 °C inactivated Pmt4, which showed an optimal activity between 20 and 25 °C (Fig. 2*F*). The optimal pH was determined to be around 7.5 (Fig. 2*G*). At concentrations between 1 and 10 mm, the divalent cations Mg^2+^, Ca^2+^, and Mn^2+^ had a beneficial effect on Pmt4 activity, but EDTA did not significantly impact on the mannosyl transfer (Fig. 2*H*).

**FIGURE 3.**
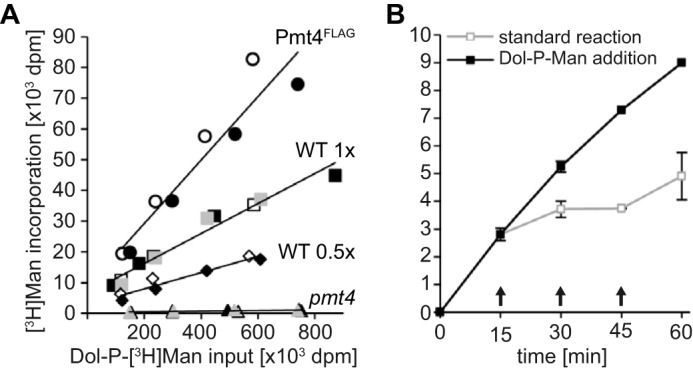
**Dependence of the Pmt4 *in vitro* mannosyltransferase activity on the Dol-P-[^3^H]Man input.** *A* and *B,* unless otherwise stated, *in vitro* mannosyltransferase activity was determined under standard conditions including 4 μg of the mannosyl acceptor GST-αDG as detailed under ”Experimental Procedures.“ *A,* crude membranes from strains SEY6210 (WT; *squares*, 0.5 μg/μl; *diamonds*, 0.25 μg/μl) and *pmt4* expressing pRS423 (*pmt4*; *triangles*, 0.5 μg/μl) or pJK4-B1 (overexpression of Pmt4^FLAG^; *circles*, 0.5 μg/μl; see also Fig. 2*B*), were used as enzyme source and *in vitro* mannosyltransfer at the indicated donor substrate concentrations was determined. Results of independent experiments are shown in different shades. *B,* SEY6210 (WT) membranes were used and *in vitro* mannosyltransfer was determined at indicated time points for the standard reaction and for fresh Dol-P-[^3^H]Man addition every 15 min (indicated by *arrows*) (*n* = 2–5). The average Dol-P-[^3^H]Man input was between 18,000 and 32,000 dpm in the standard reaction and between 23,000 to 34,000 dpm for all consecutive additions.

Increasing Dol-P-Man concentrations enhanced the *in vitro* transfer of [^3^H]mannose almost linearly (Fig. 3*A*). At all concentrations the amount of the [^3^H]mannose transferred to GST-αDG was proportional to the amount of the Pmt4 input (Fig. 3*A*, WT: 0.5 and 0.25 μg/μl of membranes) although relative incorporation levels did not increase to more than 25% (Figs. 2*A* and 3*A*). Likely explanations of this limited incorporation are the transfer of [^3^H]mannose to endogenous mannosyl acceptors by PMTs and other Dol-P-Man utilizing enzymes present in the crude membranes. This view is supported by the fact that overexpression of Pmt4^FLAG^ steepened the level of [^3^H]mannose transferred to GST-αDG (Fig. 3*A*, Pmt4^FLAG^: 0.5 μg/μl of membranes), and that additions of fresh Dol-P-Man to the standard assay in 15-min intervals linearly increased incorporation of [^3^H]mannose into GST-αDG (Fig. 3*B*).

##### Pmt4 and Pmt1-Pmt2 Have Distinct Detergent and Acceptor Substrate Requirements in Vitro

**FIGURE 4.**
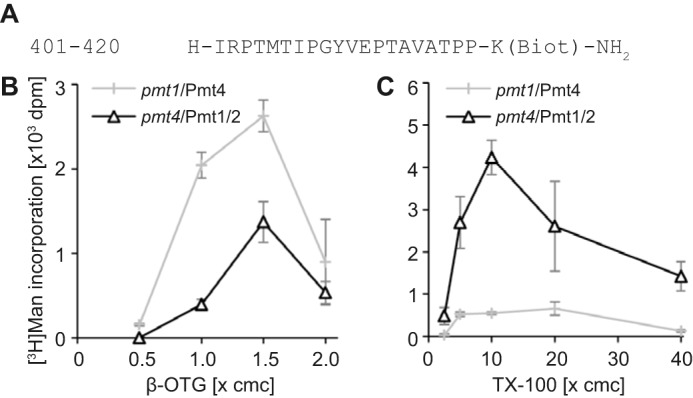
**Detergent requirements of Pmt4 and Pmt1–2 complexes.** *In vitro O*-mannosyltransferase activity was determined as detailed under ”Experimental Procedures.“ Mean ± S.D. values of three replicates are shown. *A,* amino acid sequence of peptide 401–420-bio. *B* and *C,* dependence of mannosyltransferase activity on detergent concentrations for β-OTG (*B*) or Triton X-100 (*C*) is shown for Pmt4 (enzyme source: membranes from a *pmt1* deletion strain; *pmt1*/Pmt4) and for Pmt1-Pmt2 complexes (enzyme source: membranes from a *pmt4* deletion strain; *pmt4*/Pmt1/2) using the 401–420-bio peptide. Detergent concentrations are indicated as a function of the critical micelle concentration (*cmc*). The average Dol-P-[^3^H]Man input was 23,357 (*B*) and 33,225 dpm (*C*) per reaction.

Our analysis revealed that in the presence of β-OTG as a detergent yeast Pmt4 can mannosylate the mammalian POMT substrate GST-αDG (Fig. 2*A*). In a *pmt4*Δ mutant, other *O-*mannosyltransferase activities were below the detection limits of the assay (Fig. 2*A*), although the Pmt1-Pmt2 complex is fully active *in vitro* in the absence of Pmt4 (with Triton X-100 as detergent) (8). In contrast, our previous studies showed *in vitro* mannosylation of the acceptor peptide bio-YATAV by Pmt1-Pmt2, but not by Pmt4, in the presence of the detergent Triton X-100 (17, 32). To further address *in vitro* mannosyl acceptor specificities of the yeast PMT family members, we first analyzed the detergent requirements in more detail. To individually record endogenous Pmt4 and Pmt1-Pmt2 activities, membranes from *pmt1* and *pmt4* deletion mutants, respectively, were used as an enzyme source. For vivid depiction, membranes from these strains are identified as *pmt1*/Pmt4 and *pmt4*/Pmt1/2 in Table 1 and Fig. 4. Under the conditions applied, in mutant *pmt1*Δ *in vitro* enzymatic activity of Pmt2 and other PMT1 and PMT2 family members is negligibly small (17). Even in the presence of Triton X-100, GST-αDG did not serve as acceptor substrate of Pmt1-Pmt2 (data not shown). But, a proven POMT *in vitro* substrate, the αDG-derived synthetic peptide including amino acids 401 to 420 (29) (Fig. 4*A*) qualified as a more general mannosyl acceptor. Following the transfer of [^3^H]mannose from Dol-P-Man to peptide 401–420-bio showed that Pmt4 and Pmt1-Pmt2 complexes were both active with β-OTG as detergent, and showed similar β-OTG optima at ∼1.5 × critical micelle concentration (∼0.42%). Although protein levels of Pmt4 are at least 2–3 times lower when compared with Pmt1 and Pmt2 (data not shown), Pmt4 activity was significantly higher (Fig. 4*B*). In contrast, Pmt4 was almost inactive with Triton X-100 as detergent, whereas Pmt1-Pmt2 activity was characterized by an optimal curve with the highest [^3^H]mannose transfer at ∼0.12% Triton X-100 (∼10 × critical micelle concentration; Fig. 4*C*).

**TABLE 1:** **Substrate preferences of Pmt4 and Pmt1-Pmt2 complexes** *In vitro* mannosyltransferase activity was determined as detailed under ”Experimental Procedures.“ Mean ± S.D. values of three replicates are shown. Substrate specificities of endogenous Pmt4 and Pmt1-Pmt2 complexes in comparison to SEY6210 (WT) were determined using membranes from *pmt1* (*pmt1*/Pmt4) and *pmt4* (*pmt4*/Pmt1/2) deletion strains, respectively. Assays were performed using different biotinylated acceptor peptides (bio-YATAV, 401–420-bio, and 418–440-bio) and standard reaction conditions with β-OTG as detergent. The average Dol-P-[^3^H]Man input was 49,936 dpm per reaction for 401–420-bio and 418–440-bio peptides and 39,323 dpm per reaction for the bio-YATAV peptide.

Peptide	Sequence	Strain	[^3^H]Man incorporation (×10^3^ dpm) ± S.D.	% ± S.D.
YATAV	(Biot)-NH-YATAV-CONH_2_	WT	1.516 ± 0.191	100 ± 13
		*pmt1*/Pmt4	0.027 ± 0.016	2 ± 1
		*pmt4*/Pmt1/2	2.381 ± 0.483	157 ± 32
401–420	H-IRPTMTIPGYVEPTAVATPP-K(Biot)-NH_2_	WT	5.828 ± 0.369	100 ± 6
		*pmt1*/Pmt4	4.095 ± 0.745	70 ± 13
		*pmt4*/Pmt1/2	2.022 ± 0.378	35 ± 6
418–440	H-TPPTTTTKKPRVSTPKPATPSTD-K(Biot)-NH_2_	WT	1.830 ± 0.295	100 ± 16
		*pmt1*/Pmt4	0.500 ± 0.180	27 ± 10
		*pmt4*/Pmt1/2	1.047 ± 0.078	57 ± 4

Because both Pmt4 and Pmt1-Pmt2 activities could be monitored in the presence of β-OTG (Fig. 4*B*), *in vitro* substrate preferences were further determined in standard reactions including this detergent at 1.5 × critical micelle concentration (detailed under “Experimental Procedures”). Even with β-OTG, peptide bio-YATAV was only mannosylated by Pmt1-Pmt2 (Table 1). In line with previous observations, Pmt1-Pmt2 *in vitro* activity was enhanced ∼1.5-fold in the absence of Pmt4 (Table 1) (32). In contrast to bio-YATAV, the αDG-derived acceptor peptides 401–420-bio and 418–440-bio (29) served as mannosyl acceptors for Pmt4 and Pmt1-Pmt2, although to differing degrees (Table 1). Peptide 401–420-bio (four putative *O*-mannosyl acceptor sites) was preferentially mannosylated by Pmt4 (∼70% of the mannosyl transfer activity in wild-type membranes; Table 1), whereas the poor POMT substrate 418–440-bio (ten putative *O*-mannosyl acceptor sites; 29), which was also a weak substrate for yeast PMTs, was favored by Pmt1-Pmt2 (∼57% of the mannosyl transfer activity in wild-type membranes; Table 1).

##### Yeast Pmt4 and Human POMTs Have Similar Preferences toward Mannosyl Acceptor Peptides in Vitro

**FIGURE 5.**
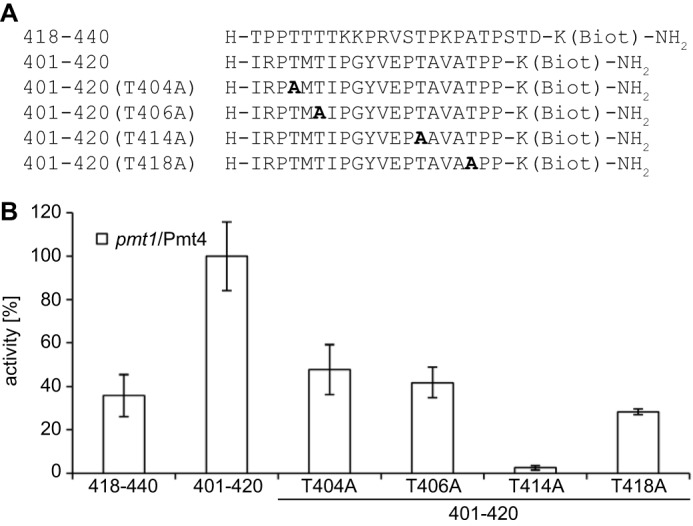
**Mannosyl acceptor peptide preferences of Pmt4.** *In vitro O*-mannosyltransferase activity was determined as described under ”Experimental Procedures.“ Mean ± S.D. values of three replicates are shown. *A,* peptide sequences of the mannosyl acceptors 418–440-bio, 401–420-bio, and Thr to Ala mutations thereof. *B,* mannosyltransferase activity of endogenous Pmt4 was measured using crude membranes from a *pmt1* deletion mutant (*pmt1*/Pmt4). Standard reaction conditions were applied for the indicated acceptor peptides. Relative activities are displayed with respect to the peptide 401–420-bio for which activity was set to 100%. The average Dol-P-[^3^H]Man input was 33,314 dpm/reaction.

Our analysis revealed that *in vitro* properties of Pmt4 resemble those from human POMTs, but show some distinct features when compared with yeast Pmt1-Pmt2. To further explore similarities in acceptor preferences between POMTs and yeast Pmt4, *in vitro O*-mannosylation of peptide 401–420-bio-derived variants in which the four putative mannosylation sites were individually exchanged to alanine were analyzed (Fig. 5*A*). It was previously reported for the human POMT complex that changing Thr-404, Thr-406, and Thr-414 to Ala greatly reduced acceptor efficiency of peptide 401–420 (29). As shown in Fig. 5*B*, Pmt4-based transfer of [^3^H]mannose to peptides T404A, T406A, T414A, and T418A was significantly decreased with T414A showing the most pronounced effect (∼2% acceptor efficiency) (Fig. 5*B*).

##### Impact of POMT1 Mutations on Pmt4 in Vitro Mannosyltransferase Activity

Our experiments showed that *in vitro*, Pmt4 and POMTs recognize the same αDG-derived acceptor substrates and have similar detergent requirements. These findings prompted us to analyze whether dystroglycanopathy-associated mutations in human POMT1 also affect enzymatic activity of yeast Pmt4. We chose the point mutations G76R and V428D that had originally been detected in *POMT1* of WWS patients (13), and created the corresponding Pmt4 mutants I112R and I435D. The exchanged amino acids are not highly conserved between POMT1 and Pmt4, however, that position is never occupied by a charged residue (Fig. 1). According to the topology model of PMTs (18), mutation G76R/I112R locates to the conserved loop1 region (Fig. 1). In Pmt1, this loop has been recently implicated in acceptor substrate binding (19). Mutation V428D/I435D is situated within a moderately conserved stretch of the second MIR motif within loop5 (Fig. 1). As a control, Pmt4 Ile-435 was changed to valine (I435V), which mimics the wild-type POMT1 allele (Fig. 1).

**FIGURE 6.**
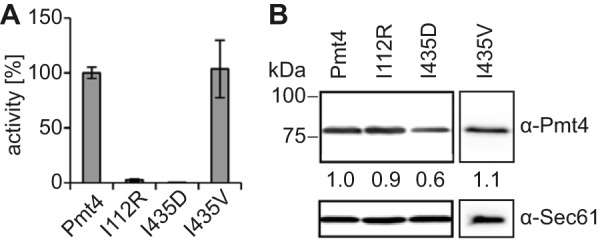
***In vitro* mannosyltransferase activity of Pmt4 mutant proteins.** *A, in vitro* activities of Pmt4 and the variants thereof. *In vitro* mannosyltransferase activity was determined as specified under ”Experimental Procedures.“ Mean ± S.D. values of at least three independent experiments are shown as relative activities referring to Pmt4^FLAG^ for which activity was set to 100%. Crude membranes from *pmt4* null mutants expressing pJK4-B1 (Pmt4), pMS1 (I112R), pMS2 (I435D), or pDB6 (I435V) were used as enzyme source. The average Dol-P-[^3^H]Man input was 33,942 dpm/reaction for Pmt4, I112R, and I435D or 29,758 dpm/reaction for I435V. Maximal activities of about 7,000 dpm could be measured for wild-type Pmt4 with background values of around 100 dpm when no acceptor was added. Thus, activities below 1.5% of WT could not be evaluated. *B,* Western blotting analysis based quantification of Pmt4 variants in the membrane preparations which were used in *A*. Blots were probed with anti-Pmt4 and anti-Sec61 antibodies. Signal intensities were detected and analyzed as outlined under ”Experimental Procedures.“ The indicated numbers represent the relative Pmt4 content (ratio Pmt4:Sec61) with reference to wild-type Pmt4, which was set as 1.

Under standard conditions with GST-αDG as mannosyl acceptor substrate, mannosyltransferase activity of the different enzyme variants was analyzed. Crude membranes were hence prepared from a *pmt4* null mutant expressing FLAG-tagged versions of either wild-type or mutant Pmt4. Western blotting analysis revealed that the steady-state levels of Pmt4^FLAG^ and mutants I112R, I435D, and I435V did not significantly differ from each other (Fig. 6*B*). In these preparations, mannosyltransferase activity of mutant I112R was highly reduced (by ∼97.5% when compared with the quantifiable activity of the wild-type enzyme). Enzymatic activity of mutant I435D could not be detected within the limits of the assay, whereas mutant I435V was at wild-type level (Fig. 6*A*).

##### Impact of POMT1 Mutations on Pmt4 in Vivo Mannosyltransferase Activity

**FIGURE 7.**
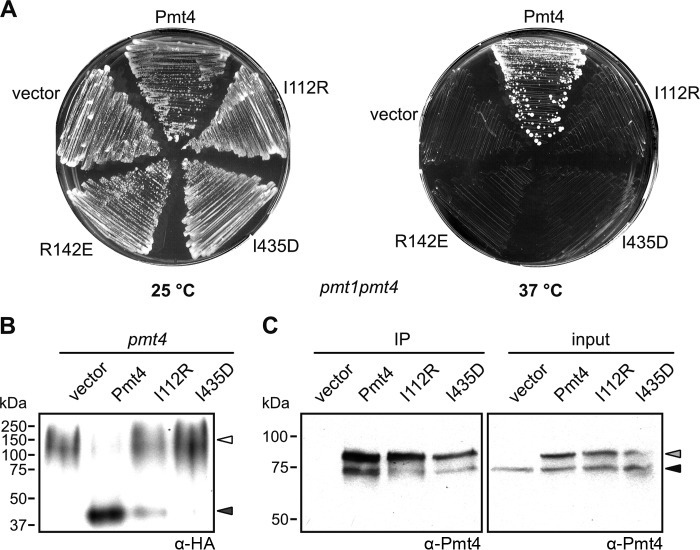
**Functionality of Pmt4 mutants *in vivo*.** *A,* complementation of the temperature-sensitive phenotype of strain *pmt1pmt4*. The double deletion strain *pmt1pmt4* was transformed with plasmids pRS423 (vector), pJK4-B1 (Pmt4), pMS1 (I112R), pMS2 (I435D), and pVG45 (R142E). Strains were grown at 25 °C (*left*) and 37 °C (*right*) for 3 days. *B,* Pmt4-dependent glycosylation of the cell wall protein Ccw5. Yeast mutant *pmt4* expressing HA-tagged Ccw5 from plasmid pCCW5-HA was transformed with the plasmids pRS423 (vector), pJK4-B1 (Pmt4), pMS1 (I112R), or pMS2 (I435D). Ccw5^HA^ was isolated from the indicated strains as described under ”Experimental Procedures.“ *O*-Mannosylated (*dark gray arrowhead*) and *N*-glycosylated (*white arrowhead*) isoforms were monitored based on their different electrophoretic mobility during SDS-PAGE. Western blotting which was probed with anti-HA antibodies. *C,* formation of homomeric Pmt4 complexes. Protein extracts from wild-type strain SEY6210 transformed with pRS423 (vector) or plasmids encoding FLAG-tagged Pmt4 variants (pJK4-B1 (Pmt4), pMS1 (I112R), and pMS2 (I435D)) were prepared and immunoprecipitation using anti-FLAG antibodies was performed as outlined under ”Experimental Procedures.“ Immunoprecipitates (*IP*) and aliquots of the corresponding input material were analyzed by SDS-PAGE and Western blotting analysis using anti-Pmt4 antibodies. FLAG-tagged Pmt4 variants and endogenous Pmt4 are highlighted with *light gray* and *black arrowheads*, respectively.

We further examined whether the observed loss of the *in vitro* activity of mutants I112R and I435D also reflects the *in vivo* situation. An indicator of yeast Pmt4 functionality *in vivo* is the synthetic temperature sensitivity of the double deletion strain *pmt1pmt4*. This strain fails to grow at 37 °C unless a functional variant of Pmt4 is expressed (8). As shown in Fig. 7*A*, *pmt1pmt4* transformed with either the empty vector or plasmids expressing FLAG-tagged Pmt4 and variants thereof (the inactive Pmt4 mutant R142E (22) served as negative control) were viable at 25 °C but only wild-type Pmt4 restored thermotolerance of the *pmt1pmt4* strain (Fig. 7*A*), indicating that Pmt4 mutants I112R and I435D have indeed very low or no mannosyltransferase activity.

In addition, we took advantage of the fact that the *pmt4* null mutant strain displays distinct molecular features, *e.g.* aberrant glycosylation of the cell wall protein Ccw5 (33). This protein harbors an *N*-glycosylation sequon (^114^NAT^116^) situated in a region that is *O*-mannosylated by Pmt4 in the wild-type, whereby *N*-glycosylation of Asn-114 is prevented. Loss of *O*-mannosylation by Pmt4, however, results in the addition of an *N*-linked glycan at that position and, as a consequence, a shift of the apparent molecular mass of the mature Ccw5 protein from 40 to ∼100–250 kDa (33) (Fig. 7*B*). Ccw5 glycosylation as reflected by its electrophoretic mobility can therefore serve as an indicator for Pmt4 functionality *in vivo*. We hence analyzed the glycosylation status of Ccw5 isolated from cell walls of a *pmt4* deletion strain expressing wild-type or mutant Pmt4 by Western blotting. As evident from Fig. 7*B*, the glycosylation pattern of Ccw5 isolated from mutants I112R and I435D and from the *pmt4* null mutant are highly similar (∼100–250 kDa) demonstrating that enzyme function *in vivo* is largely lost in both mutants. In agreement with the *in vitro* data, a small amount of the 40-kDa form of Ccw5 could be detected in the I112R mutant, pointing to a minor residual enzymatic activity of this Pmt4 variant.

Assembly into homomeric complexes is a prerequisite for Pmt4 activity (22). To test whether the loss of the enzymatic activity of mutants I112R and I435D resulted from deficient complex formation, we performed co-immunoprecipitation experiments. For that purpose, FLAG-tagged Pmt4 variants were expressed in wild-type yeast containing endogenous Pmt4. Proteins were co-precipitated by anti-FLAG antibodies and analyzed by Western blottings which were probed with polyclonal anti-Pmt4 antibodies (Fig. 7*C*). Endogenous Pmt4 co-purified with mutant proteins I112R and I435D showing that complex formation is not grossly affected by these amino acid substitutions even though minor effects cannot be ruled out entirely.

## Discussion

Although assays for the monitoring of yeast PMT activity *in vitro* have been described already in the early 1970s (34), it became clear, along with the discovery and characterization of the corresponding enzymes, that these assays, which use small peptide acceptors, detect only a specific subset of the PMT enzymes (32). The reason why Pmt1-Pmt2 complexes act on a variety of short peptides such as the pentapeptide YATAV, whereas Pmt4 as well as the mammalian POMT1-POMT2 complex do not, is unclear to date. The lack of an adequate substrate has hindered attempts to develop an *in vitro* assay system for Pmt4 activity. Here we show that the mammalian *O*-mannosylation substrate αDG is suitable for the specific detection of yeast Pmt4 activity. This finding allows monitoring enzymatic activity of homomeric Pmt4 complexes *in vitro*.

Our analyses revealed that yeast Pmt1-Pmt2, Pmt4, and mammalian POMT1-POMT2 enzymes show various similarities *in vitro* such as pH and temperature optima, stimulation by divalent cations, and resistance to EDTA (Fig. 2) (17, 31, 35). However, their detergent requirements differ substantially. Pmt1-Pmt2 complexes are active *in vitro* in a wide range of detergents (Fig. 4) (17, 35), whereas Pmt4 and mammalian POMT *in vitro* activities largely depend on β-OTG even though numerous alternative detergents have been tested (Fig. 4 and data not shown) (27, 31). In addition, unlike Pmt1-Pmt2, Pmt4 and POMTs mannosylate GST-αDG *in vitro* (Fig. 2*A*) (31). Why the mucin domain of αDG does not serve as an acceptor substrate of Pmt1-Pmt2 remains unclear. This issue is especially puzzling because αDG-derived peptides are *in vitro* mannosyl acceptors for all PMTs, albeit with different preferences (Table 1). One possible explanation could be that in the 171-amino acid long αDG domain, which is rich in proline residues (roughly 20% overall content) and harbors a Ser/Thr content of nearly 30%, Pmt1-Pmt2 acceptor sites are masked. A putatively unstructured polypeptide containing numerous acceptor sites, however, might be sufficient to trigger *O*-mannosylation by Pmt4, at least *in vitro*. Recently, the yeast *O*-mannose glycoproteome revealed general characteristics of *O*-mannosylation sites but sequence features suggestive of a glycosylation motif did not become evident. Yeast *O*-mannosyl glycans are enriched in unstructured regions and β-strand folds that might be attributed to the discrete substrate and/or glycosylation site specificities of the different yeast PMT family members (25).

Yeast Pmt4 and POMTs do not only act on the same αDG-derived protein substrate, they also show similar mannosyl acceptor preferences. A previous study by Manya and co-workers (29) identified the αDG-derived peptide 401–420 as a POMT *in vitro* substrate, which is most frequently mannosylated at Thr-414 (T414A substitution decreased the mannosyl transfer by 93%), and mannosylation of this Thr residue most likely facilitates subsequent modification by the POMT complex. In good agreement with these data, peptide 401–420-bio is a preferred *in vitro* substrate of yeast Pmt4 (Table 1, Figs. 4 and 5). Although *O*-mannosylation site occupancy has not been directly analyzed by mass spectrometry, individual substitutions of the Thr residues in this peptide for Ala indicate that all four hydroxy amino acids serve as acceptor sites of Pmt4, and that Thr-414 is especially crucial for acceptor efficiency (decrease of mannosyl transfer by 98%, Fig. 5*B*). Furthermore, mannosylation effectiveness of Thr-404 and Thr-406 is comparable between Pmt4 (Fig. 5*B*) and POMTs (∼20–30%; 29). Nevertheless, Thr-418 was only poorly mannosylated by POMTs (29) but serves as a mannosyl acceptor of Pmt4 (Fig. 5*B*), indicating also distinct differences in acceptor selection. *In vivo* Pmt4 favors membrane-bound substrates (24). Although in contrast to the Pmt4 properties *in vitro*, this feature is in agreement with the notion that virtually all described *in vivo* substrate proteins of the mammalian POMTs are membrane-associated (7 and references therein). Consistently, the acceptor selection of the mammalian POMT complex differs *in vivo* and *in vitro*. Although the αDG-derived peptide 401–420 serves as a POMT acceptor substrate *in vitro*, *in vivo* domains upstream of the actual acceptor sites are additionally required for *O*-mannosylation (29, 30).

Taken together, our findings demonstrate functional similarities between Pmt4 and the mammalian POMTs and distinguish them from the fungal Pmt1-Pmt2 family members that have distinct detergent requirements and acceptor substrates *in vitro* (Fig. 4, Table 1), and mannosylate both, soluble and membrane proteins *in vivo* (reviewed in Ref. 1). Growing evidence suggests the involvement of yeast Pmt1-Pmt2, but not Pmt4, in a novel ER quality control system (reviewed in Ref. 2), further emphasizing their differences.

Pmt4 is the closest phylogenetic relative of mammalian POMT1, which acts in a heteromeric complex with POMT2 (27). Akasaka-Manya and co-workers (28) recently suggested that POMT1 and POMT2 might fulfill discrete functions, because mutations of conserved amino acids differentially affect enzymatic activity of the complex. Exchanging a single amino acid of the conserved Asp-Glu (DE) motif in loop1 (Fig. 1) with Ala in POMT1 (E44A) resulted in the loss of *in vitro* activity of the complex, whereas the corresponding mutation in POMT2 (E86A) only slightly affected mannosyltransferase activity. Intriguingly, the corresponding mutation also rendered yeast Pmt4 (E81A) inactive but only moderately affected Pmt1 (E78A) (19). Although functional similarities of mammalian and yeast PMT4 family members exist, heterologous expression of human POMT1 and POMT2 separately or in combination did not result in the complementation of the temperature-sensitive phenotype of *S. cerevisiae* double mutant *pmt1pmt4* (data not shown). Furthermore, POMT1 and POMT2 did not rescue the lethality of *Schizosaccharomyces pombe* mutant *oma2*Δ (9).^7^ This may be for several reasons including number and nature of the substrate proteins and/or impaired association of human POMTs with the yeast Sec61 translocon, which has been recently demonstrated for *S. cerevisiae* PMTs (36). The findings are in line with our previous observations that even between *S. pombe* and *S. cerevisiae* PMTs are only partially functional interchangeably (9).^7^

POMT mutations are frequently associated with congenital muscular dystrophies with widely varying clinical phenotype (13, 37–39). We and others showed that the degree of severity of the disease of patients with POMT1 mutations is inversely proportional to the POMT *in vivo* and *in vitro* activity (16, 40). Here, we mimicked two WWS-associated POMT1 amino acid exchanges in yeast Pmt4. The Pmt4 mutants I112R and I435D presented to a large extent proteolytically stable and properly folded as judged from complex formation (Figs. 6*B* and 7*C*). Yeast mutants proved catalytically inactive *in vitro* and *in vivo* (Figs. 6*A* and 7), which is consistent with the severe phenotypes of the patients in which the corresponding POMT1 mutations had been discovered and the highly reduced POMT *in vitro* activity of fibroblasts derived from a WWS patient carrying the homozygous mutation G76R (13, 16).

Without structural models it is difficult to judge how the analyzed mutations affect PMT/POMT activity. The loop5 domain is highly homologous to the ER-resident soluble stromal cell-derived factor 2 (SDF2) (21). Recently, we resolved the three-dimensional crystal structure of *Arabidopsis thaliana* SDF2 at 1.95-Å resolution that revealed the typical β-trefoil fold and consists of 12 β-strands and three 3_10_ helices forming a globular barrel (41). Conserved leucine and valine residues form hydrophobic layers of the barrel with a crucial role in maintaining the β-trefoil structure (41). To further address the impact of the analyzed Pmt4 I435D mutation, based on *At*SDF2 we generated structural models of the yeast Pmt4-, Pmt1-, and human POMT1-loop5 domains (supplemental Fig. S1). Like in *At*SDF2, the β-trefoil-fold of loop5 is made up of three structural repeats that correspond to the three MIR motifs, giving rise to a pseudo 3-fold symmetry. Pmt4 Ile-435 and POMT1 Val-428 are part of a hydrophobic layer at the top of the bottom layer of the barrel (supplemental Fig. S1). Thus, mutations Pmt4 I435D and POMT1 V428D almost certainly interfere with the general structure of the respective loop5 domain. Similarly, exchange of a conserved leucine residue of the hydrophobic layer of Pmt1-loop5 (L408A) also affects enzymatic function (supplemental Fig. S1) (20), suggesting that the β-trefoil-fold of loop5 is a key feature of all PMTs/POMTs.

In summary, we set up a robust *in vitro* assay that allows the quantitative determination of Pmt4 activity. With this tool in hand, studies elucidating the fundamentals of protein *O*-mannosyltransferases are now simplified due to the amenability of a homomeric protein complex. In particular, using Pmt4 as a model will greatly facilitate structural analyses such as three-dimensional crystallization.

## Experimental Procedures

### 

#### 

##### Yeast Strains and Plasmids

The *S. cerevisiae* disruptants *pmt4* (*pmt4*::*TRP1*) (32), *pmt1* (*pmt1*::*HIS3*) (42), and *pmt1pmt4* (*pmt1*::*URA3*, *pmt4*::*TRP1*) (8) are descendants of the reference strain SEY6210 (*MAT*α, *his3*-Δ*200, leu2–3,* −*112, lys2–801, trp1*-Δ*901, ura3–52, suc2*-Δ*9*) (43). Yeasts were grown under standard conditions and transformed according to Hill *et al.* (44) with plasmids pJK4-B1 (PMT4^FLAG^), pVG45 (PMT4-R142E^FLAG^) (22), and pCCW5-HA(33) and the plasmids described below. *PMT4* point mutations were introduced into pJK4-B1 via site-directed mutagenesis using recombinant PCR (45). Sequences of the oligonucleotides used in this study are available upon request. DNA constructs were processed using standard procedures and routinely verified by sequence analysis.

To create plasmid pMS1 (PMT4-I112R^FLAG^), a PCR fragment generated with the mutagenic primer pair 510/511 in combination with the outer primers vg28 and 512, was subcloned into pJK4-B1 via PaeI and Van91I. Plasmid pMS2 (PMT4-I435D^FLAG^) was generated with the mutagenic primer pair 508/509 and outer primers vg28 and vg27. The resulting fragment was subcloned into pJK4-B1 via Van91I and KspAI. Plasmid pDB6 (PMT4-I435V^FLAG^) was assembled by generating a PCR fragment with the mutagenic primer pair 2485/2486 and the outer primers vg28 and 512 and subcloning the resulting fragment into pJK4-B1 linearized with NcoI and EcoNI via homologous recombination in yeast.

##### Immunoprecipitation

Immunoprecipitation experiments were performed as previously described (22).

##### Preparation of Crude Membranes and Cell Wall Extracts from S. cerevisiae

Crude yeast membranes were prepared essentially as previously described (20) with minor modifications. Briefly, exponentially growing yeast cells were harvested by centrifugation, washed once with water, and once with 50 mm Tris-HCl, pH 7.4, 5 mm MgCl_2_. The pellet was resuspended in the same buffer plus 1 mm PMSF, 1 mm benzamidine, 0.25 mm 1-chloro-3-tosylamido-7-amino-2-heptanone, 50 μg/ml of l-1-tosylamido-2-phenylethyl chloromethyl ketone, 10 μg/ml of antipain, 1 μg/ml of leupeptin, and 1 μg/ml of pepstatin. An equal volume of glass beads was added and cells were lysed in a Hybaid RiboLyser (4 × 25 s at level 4.5 with 1-min intervals on ice) at 4 °C. The bottom of the tube was punctured, and the lysate was collected into a new tube. Cell debris was removed by two successive centrifugations (5 min at 1,500 × *g*, 4 °C). The supernatant was then centrifuged 1 h at 20,000 × *g* at 4 °C. Pelleted membranes were resuspended in 20 mm Tris-HCl, pH 8.0, 10 mm EDTA, 15% (v/v) glycerol plus protease inhibitors (see above), frozen in liquid nitrogen, and stored at −80 °C. Cell wall extracts were prepared as described in Ref. 33.

##### Preparation of GST-tagged αDG Mucin Domain

GST-αDG and GST were purified from *Escherichia coli* BL21(DE3) cells. The corresponding expression plasmids and the purification procedure are described in Ref. 46.

##### Biotinylated Peptide Acceptors

401–420-bio and bio-YATAV were purchased from Biopolymers Thermo Scientific, 418–440-bio and Thr to Ala substitutions of 401–420-bio from Intavis AG.

##### Pmt4 Specific in Vitro Mannosyltransferase Activity Assay

Pmt4 *in vitro* activity measurements were based on the previously published protocol by Manya and co-workers (31) with minor modifications. Standard reactions contained 20,000–40,000 dpm (150–300 fmol) of [^3^H]mannose-labeled Dol-P-Man (mannosylphosphoryldolichol-95, American Radiolabled Chemicals, 60 Ci/mmol), 4 μg of acceptor protein (GST-αDG or GST), 30 μg of total protein of crude membrane preparations, 0.45% (w/v) β-OTG, 2 mm β-mercaptoethanol, 10 mm EDTA, and 20 mm Tris-HCl, pH 7.5, in a total reaction volume of 20 μl. Prior to the reaction, Dol-P-Man was dried in a glass vial under a stream of nitrogen and resuspended in the reaction mixture (containing everything but the proteins) by extensive vortexing and ultrasonication. After addition of the acceptor protein, the reaction was started by the addition of membranes, incubated 15 min at 20 °C, and then stopped with 200 μl of ice-cold PBS plus 1% Triton X-100. After centrifugation for 10 min at 20,000 × *g* at 4 °C, the supernatant was incubated with GSH-Sepharose beads (GE Healthcare) for 90 min at 4 °C. The beads were washed two times with PBS plus 1% Triton X-100 and two times with PBS. Incorporated radioactivity was measured by liquid scintillation counting.

##### In Vitro Mannosyltransferase Assay Using Biotinylated Peptide Acceptors

Standard reactions were performed as described above. Instead of GST-αDG, synthetic peptides (200 μm) carrying a C-terminal Biotin tag were included as mannosyl acceptor. After stop of the reaction, mixtures were centrifuged for 10 min at 20,000 × *g* at 4 °C. The supernatant was incubated with 20 μl of slurry of High Capacity Neutravidin®-agarose (Thermo Scientific) for 1 h at 4 °C. Beads were washed two times with PBS plus 1% Triton X-100 and two times with PBS. Incorporated radioactivity was measured by liquid scintillation counting.

##### Western Blotting Analysis

Protein samples were resolved by SDS-PAGE on 8% polyacrylamide gels and transferred to nitrocellulose. Monoclonal mouse anti-FLAG (M2, Sigma) antibodies were used at a dilution of 1:5,000. Polyclonal rabbit Pmt4- (22) and Sec61-directed (47) antibodies were used at a dilution of 1:2,500 and 1:1,000, respectively. Blots were incubated with horseradish peroxidase-conjugated anti-mouse or anti-rabbit secondary antibodies (Sigma). Protein-antibody complexes were visualized by enhanced chemiluminescence and quantified with the ImageQuant LAS 4000 imaging system (GE Healthcare).

## Author Contributions

D. B. designed, performed, and analyzed the experiments shown in Figs. 4–6, supplemental Fig. S1, and Table 1. J. E. and T. J. designed, performed, and analyzed the experiments shown in Figs. 2, 3, and 6. M. S. performed and analyzed the experiments shown in Fig. 7. J. E. designed Fig. 1. S. S., J. E., and D. B. wrote the manuscript. All authors reviewed the results and approved the final version of the manuscript. S. S. conceived and coordinated the study.

## Supplementary Material

Supplemental Data

## Footnotes

7 T. Willer and S. Strahl, unpublished data.

## Footnotes

6 The abbreviations used are:

ER: endoplasmic reticulum
Dol-P-Man: dolichol monophosphate-activated mannose
αDG: α-dystroglycan
PMT: protein *O*-mannosyltransferase
WWS: Walker-Warburg syndrome
β-OTG: β-octylthioglucoside.
