# Supplementary material for: Functional Similarities between the Protein O-Mannosyltransferases Pmt4 from Bakers' Yeast and Human POMT1
Source: J Biol Chem. 2016 Jun 29;291(34):18006–15. doi: 10.1074/jbc.M116.739128 (PMC5016187; doi:10.1074/jbc.M116.739128)
Supplement: Supplemental Data [file supp_291_34_18006__index.html]

Functional similarities between the protein O-mannosyltransferases Pmt4 from baker's yeast and human POMT1 — Functional Similarities between the Protein O-Mannosyltransferases Pmt4 from Bakers' Yeast and Human POMT1 — Yeast Protein O-Mannosyltransferase Pmt4 — Supplemental Data 

# Functional Similarities between the Protein *O*-Mannosyltransferases Pmt4 from Bakers' Yeast and Human POMT1

## Supplemental Data

- Supplemental Figure S1 (.pdf, 530 KB) - Structural models of the loop5 domain of PMTs
