# Supplementary material for: Functional Similarities between the Protein O-Mannosyltransferases Pmt4 from Bakers' Yeast and Human POMT1
Source: J Biol Chem. 2016 Jun 29;291(34):18006–15. doi: 10.1074/jbc.M116.739128 (PMC5016187; doi:10.1074/jbc.M116.739128)

Functional Similarities between the Protein *O*-Mannosyltransferases Pmt4 from Baker´s Yeast and Human POMT1

Daniela Bausewein‡, Jakob Engel‡[1], Thomas Jank‡[2], Maria Schoedl[3], and Sabine Strahl[4]

SUPPLEMENTAL FIGURE S1. **Structural models of the loop5 domain of PMTs.** (*A*) Alignment of *Arabidopsis thaliana* SDF2 with loop5 regions of *Saccharomyces cerevisiae* Pmt1, Pmt4 and human POMT1. Protein sequence alignment was performed with the Clustal Omega software. BOXSHADE (version 3.21, written by K. Hofmann and M. Baron) was used to determine identical and conserved amino acids. The fraction of sequences that must agree for shading was set as 0.7. For the sake of clarity, in accordance to BOXSHADE identical and conserved amino acids are highlighted in dark and light green, respectively. Location of the 12 β-strands in SDF2 is depicted. The three MIR motifs are colored in green (α-repeat, MIR1), magenta (β-repeat, MIR2) and blue (γ-repeat, MIR3). Conserved residues at the interior of the β-trefoil barrel are boxed in lilac and cyan, key residues of the triangular cap in yellow and red. (*B*) Ribbon representation of the Pmt1-, Pmt4- and POMT1-loop5 domain structure modelled to the structure of *At*SDF2 using the SWISS-MODEL server. MIR motifs are colored as in *A*. The view from the top of the triangular cap in the lower panel is rotated by 90° along a horizontal axis compared to the view in the upper panel. In the top view conserved Leu residues in β-2, β-6 and β-10 are shown in stick representation and colored in yellow (I). In addition, Val and Ile residues including the ones homologous to Ile-435 in Pmt4 are highlighted in red (II). Models were generated with PyMOL.

Supplemental Figure S1

A

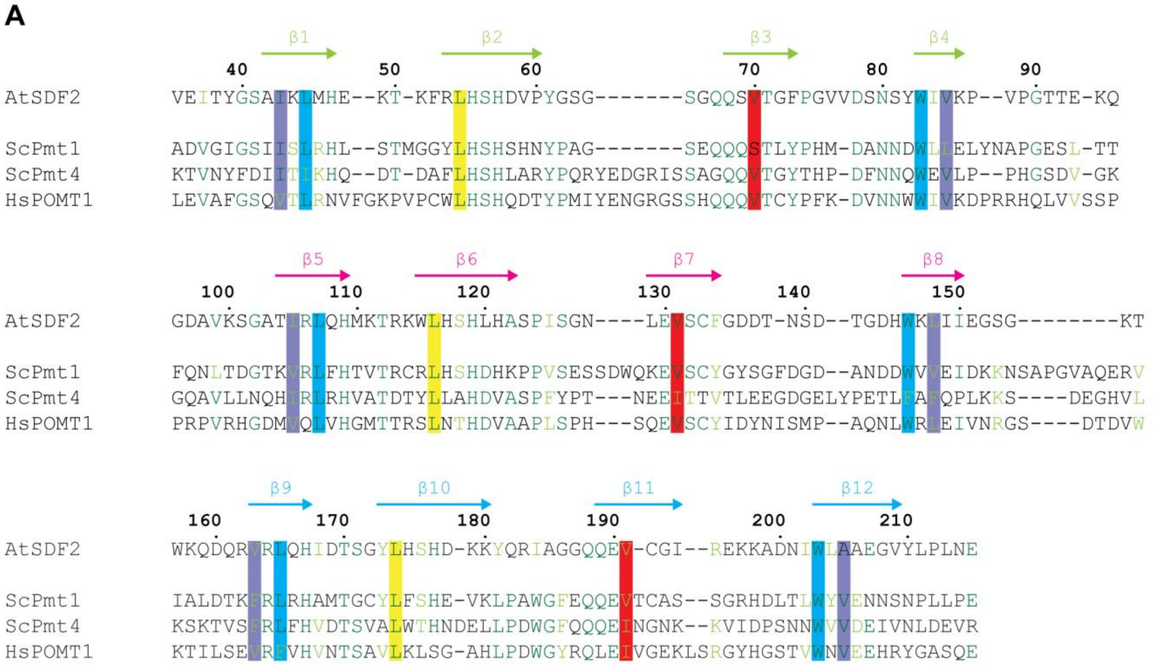

B

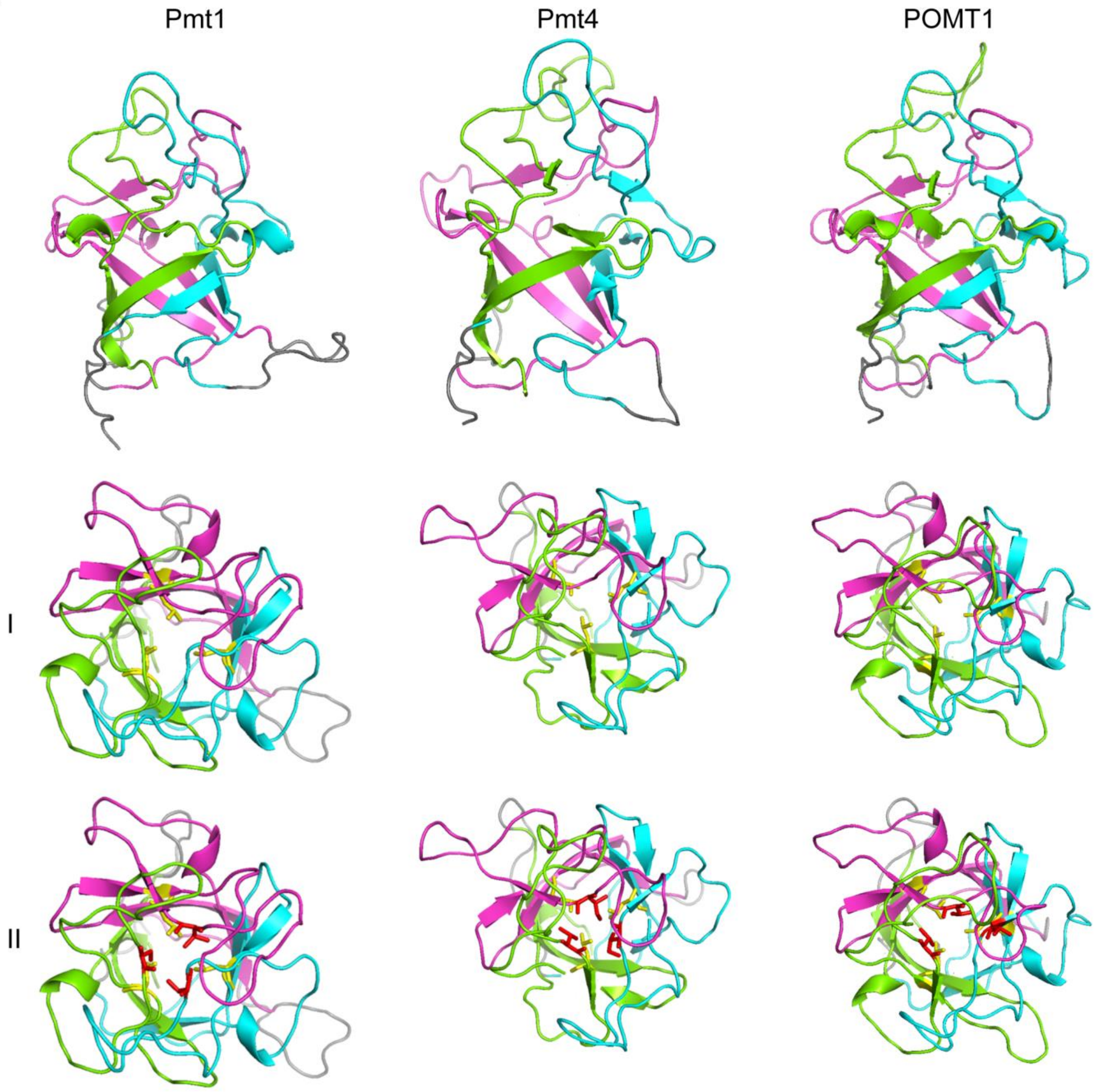

Supplement: Supplemental Data [file 10.1074_M116.739128_jbc.M116.739128-1.pdf]
